# Assessment of cerebrovascular interactions and control in coronary artery disease patients undergoing anaesthesia through bivariate predictability measures

**DOI:** 10.1007/s11517-025-03476-x

**Published:** 2025-12-02

**Authors:** Roberta Saputo, Riccardo Pernice, Laura Sparacino, Vlasta Bari, Francesca Gelpi, Alberto Porta, Luca Faes

**Affiliations:** 1 Department of Engineering, University of Palermo, Palermo, Italy; 2 Department of Biomedical Sciences for Health, University of Milan, Milan, Italy; 3 Department of Cardiothoracic, Vascular Anesthesia and Intensive Care, IRCCS Policlinico San Donato, Milan, Italy; 4 Faculty of Technical Sciences, University of Novi Sad, Novi Sad, Serbia

**Keywords:** Cerebrovascular regulation, Coronary artery bypass grafting, Anaesthesia, Cerebral blood velocity, Arterial pressure, Granger Causality, Granger Autonomy

## Abstract

Cerebrovascular regulation, driven by mechanisms such as cerebral autoregulation and the Cushing’s reflex, plays a critical role in maintaining cerebral blood flow (CBF) adequate despite changes in arterial pressure (AP), since a dampening of CBF can lead to serious brain pathologies. This study investigates the causal and self-predictable dynamics of cerebrovascular interactions in patients undergoing coronary artery bypass graft surgery, before and after propofol general anaesthesia. The dynamics of the *pressure-to-flow* and *flow-to-pressure* links between mean arterial pressure (MAP) and mean cerebral blood velocity (MCBv) is assessed using time-domain and frequency-domain measures of Granger Causality (GC) and Granger Autonomy (GA). The results indicate that while time-domain indices remain stable, frequency-domain measures reveal variations in the very-low-frequency, low-frequency, and high-frequency (HF) bands. The increased spectral GC in the HF band may be related to the effect of mechanical ventilation during anaesthesia. Additionally, a reduction in self-dependency of MCBv in the HF band reflects weakened internal regulatory mechanisms post-anaesthesia. In conclusion, propofol-induced suppression of sympathetic control and the effects of mechanical respiration increase the dependence of cerebral blood flow on arterial pressure in specific bands of cerebrovascular interest. These findings underscore the importance of frequency-domain analysis in detecting subtle cerebrovascular dynamics that time-domain measures may overlook.

**Graphical Abstract:**

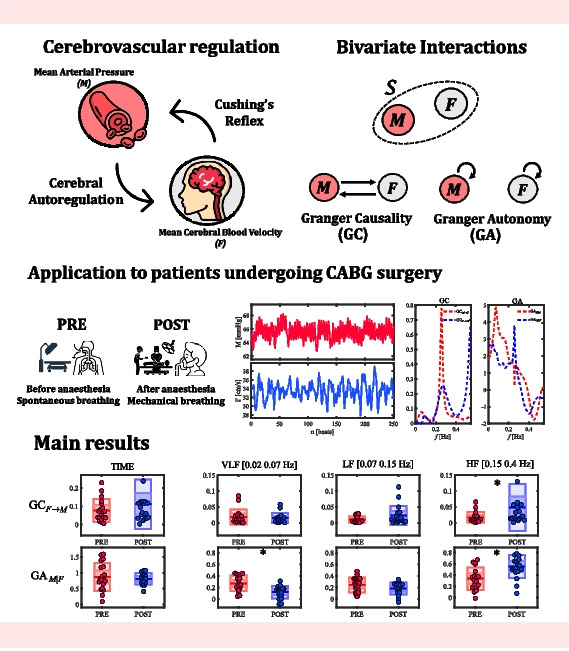

## Introduction

The brain is the most metabolically demanding organ in the human body, despite accounting for only 2% of body weight [1]. Adequate cerebral perfusion and continuous oxygen, glucose and nutrient delivery are necessary to satisfy the high metabolic demands, perform vital functions and maintain consciousness [2]. Variations in cerebral blood flow (CBF) due to spontaneous or induced stimuli, e.g., swings in arterial pressure (AP), alter cerebral homeostasis, making the brain susceptible to both conditions of hypoperfusion and hyperperfusion [3–5]. This susceptibility can lead to brain pathologies, i.e., stroke and ischemia, resulting in serious or even potentially fatal damage [6]. In this scenario, cerebrovascular regulation is fundamental in humans for the maintenance of suitable values of AP and CBF despite internal or external disturbances [6]. Two physiological mechanisms work in a complementary way to ensure brain perfusion i.e., cerebral autoregulation (CA) and Cushing’s reflex. Specifically, CA ensures the maintenance of an adequate CBF by actively counter-regulating the vessel diameter in response to AP changes within the range of 60–150 mmHg [7]. On the other hand, the Cushing’s reflex increases AP in the attempt of favouring brain perfusion in response to an acute elevation on intracranial pressure, leading to brain hypoperfusion [8]. Cushing’s reflex seems to be active even under physiological conditions to provide a fine tuning of perfusion pressure [9].

The assessment of CA can be determined by measuring the CBF response to slow (static method) or rapid (dynamic method) changes in AP [10]. Furthermore, the introduction of the transcranial Doppler (TCD) ultrasound technique allows a non-invasive assessment of dynamic CA, under the assumption that the cerebral blood velocity (CBv) can be accepted as a widely employed surrogate of CBF [11, 12], under the hypothesis that the vessel diameter is constant [13]. This approach exploits mean AP (MAP) changes induced by an external intervention, e.g., deflation of the thigh cuff, or MAP changes that occur spontaneously mainly in response to internal stimuli.

The combined action of several factors and/or confounding variables can affect cerebral autoregulation [14]. These factors are profoundly affected by the anaesthetic agents, both intravenous and volatile, at multiple levels through the alteration of arterial blood pressure, direct cerebral vasodilation, suppression of metabolism and/or of autonomic activity, and modulation of autonomic activity [15, 16]. Among the intravenous anaesthetic agents, propofol has the property of preserving CA in most cases, especially when combined with remifentanil [17]. Conversely, alteration of CA was observed when propofol is administered in high doses in patients with head trauma [16].

The role of respiration is also critical in cerebrovascular regulation, since it can operate as a confounder if conditioning to it reduces the strength of the causal relationship between MAP and CBv, or as a suppressor if the opposite situation occurs [18]. Its role can reflect the effect of mechanical ventilation with positive pressure or can be also influenced by general anaesthesia with propofol, affecting the Cushing’s reflex mechanism.

The characterization of cerebrovascular regulation has been widely investigated non-invasively by studying the closed-loop dynamic interactions between the beat-to-beat variability of mean AP (MAP) and mean CBv (MCBv) [19]. The two arms of cerebrovascular control are the *pressure-to-flow* relation linking MAP to MCBv, representative of CA mechanism, and the reverse pathway (i.e., *flow-to-pressure* link), that has been more associated to the Cushing’s reflex mechanism [20, 21]. Several methods have been developed in the previous decades to analyse the dynamics of cerebrovascular system, such as the MAP-MCBv closed-loop system transfer function [22], autoregressive index [23, 24], linear parametric autoregressive time series models [25], correlation indices, neural networks and many others. However, the absence of a gold standard promotes a challenge for clinical and medical research in finding innovative approaches that allow to investigate more directly the MAP-MCBv bidirectional interaction and at the same time the autonomous dynamics of MAP and MCBv of the closed-loop system. Recently, predictability measures for bivariate systems [26] have been developed, which can be exploited to study the dynamic interactions along the *pressure-to-flow* and the *flow-to-pressure* links. In particular, the concepts of Granger Causality (GC) and Granger Autonomy (GA) have been proven useful to investigate the role of causality patterns, i.e., interactions directed from one process to another, and self-dynamics patterns, i.e., interactions that occur internally in a process independently of its link with other processes [27].

Within this context, the present work proposes a framework for the combined assessment of causal and autonomous dynamics in cerebrovascular interactions within the pressure-flow closed-loop system. The aim of this study is to give a more in-depth assessment of the physiological mechanisms of cerebrovascular control related to the coupling between spontaneous oscillations of arterial pressure and cerebral blood flow. This is achieved by evaluating GC and GA measures in time and within the frequency bands of physiological interest [21], so as to highlight aspects in the cerebrovascular regulation mechanism that cannot be revealed using time domain measures alone. The analyses have been conducted in patients scheduled for cardiac surgery, before and after the administration of propofol general anaesthesia and during spontaneous breathing and mechanical ventilation, to also elucidate the effects of the anaesthetic agent and ventilation on cerebrovascular control and interactions. We hypothesised that cerebral autoregulation is preserved during propofol anaesthesia and that the combined effect of propofol and mechanical ventilation may influence both the internal dynamics of MCBv and MAP and their interactions in specific frequency bands.

## Materials and methods

### Experimental protocol

Eighteen patients (age: 63.8 ± 7.8 years, 1 female) undergoing coronary artery bypass graft (CABG) procedures were enrolled at the Department of Cardiothoracic, Vascular Anaesthesia and Intensive Care of IRCCS Policlinico San Donato, San Donato Milanese, Milan, Italy (study registered at clinicaltrials.gov, no. NCT03169608) [28]. According to the ethical principles of the Declaration of Helsinki for medical studies involving human subjects, patients were required to sign an informed consent to join the experimental study approved by the ethical committee of San Raffaele Hospital, Milan, Italy. Entry criteria for the selection of the study population were: sinus rhythm, age > 18 years, left ventricular ejection fraction ≥ 40%, absence of pathologies affecting brain or autonomic nervous system. Patients undergoing emergency surgery were excluded from the research study. A large percentage (i.e., 79%) of these subjects had a history of hypertension. Further details on the demographic and preoperative profile of the patient population are given in Table 1.

**Table 1:** Demographic and preoperative profile of the patient population. Values indicate either the no. of patients exhibiting the given variable or the mean ± SD of the measured variable across subjects

Variable	Value
Subjects	18
Sex (males)	17
Age, years	63.8 ± 7.8
Weight, kg	80.8 ± 15.4
Height, cm	171.7 ± 8.7
Obesity (body mass index > 30)	4

The experimental protocol included two conditions: (i) ahead of surgery before the induction of general anaesthesia (*PRE*); (ii) during general anaesthesia, after intubation of the trachea and after the beginning of mechanical ventilation, before surgical opening of the chest (*POST*). During the *PRE* session, patients were breathing spontaneously, whereas during the *POST* session they were mechanically ventilated with a rate of 12–16 breaths/min. Mechanical ventilation was administered according to a volume-controlled mode.

The *PRE* session was recorded after application of standard premedications including intramuscular administration of atropine (0.5 mg) and fentanyl (100 µg). Anaesthesia was induced by the intravenous administration of propofol as a hypnotic agent, and remifentanil, as analgesic agent. The intravenous bolus injection of propofol was maintained at 1.5 mg/kg with continuous infusion at 3 mg·kg^−1^ ·h^−1^. The range of administration of remifentanil was from 0.05 to 0.5 µg·kg^−1^ ·min^−1^ (0.32 ± 0.11 µg·kg^−1^ ·min^−1^, mean ± SD). The *PRE* session was recorded 15 min before the induction of the general anaesthesia. During the *POST* session, the patients inhaled a mixture of air and oxygen (1:1) provided by a closed breathing system (fresh gas flow of 3 l/min oxygen and 3 l/min air). The *POST* session was recorded when the target plasma concentration of propofol was expected to be around 3 µg/ml based on the pharmacokinetic properties of the drug. For both *PRE* and *POST* conditions, the data acquisition lasted about 6 min [28].

#### Signal acquisition and time series extraction

Lead-II electrocardiogram (*ECG*), derived by surface electrodes, and arterial pressure (*AP*), invasively derived from a catheter inserted into the radial artery, were recorded and acquired with an analog-to-digital board (National Instruments, Austin, TX, USA) connected to a laptop, synchronously with cerebral blood velocity (*CBv*) signal derived via a transcranial Doppler ultrasound device (Multi-Dop X, DWL, San Juan Capistrano, CA, USA) from the middle cerebral artery. All signals were sampled at a frequency of 1 kHz. Further details on the experimental protocol and signal acquisition can be found in [29].

Starting from the acquired signals, stationary beat-to-beat variability time series of *N* = 250 beats were extracted for each subject in both experimental conditions (*PRE*, *POST*). The *i*-th heart period (HP) value was computed as the temporal distance between two consecutive R-wave peaks [*i*-th and (*i* + 1) peaks, with *i* being the heart rate counter] of the ECG signal. The MAP and MCBv time series were computed respectively integrating the waveform of the sampled AP and CBv signals within each detected diastolic pulse interval (i.e., the time interval between two consecutive minimum AP or CBv values), divided by the duration of the interval itself. The first diastolic point for calculating either the *i*-th MAP and the *i*-th MCBv were taken within the *i*-th HP, HP(*i*). All series were manually checked, and ectopic beats or misdetections were corrected via linear interpolation. Corrections were less than 5% of the total series length. Further details on time series extraction can be found in [29].

In the following, the beat-to-beat variability time series of HP, MAP, and MCBv will be labelled as *H*, *M* and *F*, respectively.

### Bivariate analysis of cerebrovascular interactions based on parametric estimator

To assess the pairwise interactions between physiological time series of MAP and MCBv, a linear parametric autoregressive (AR) approach formulated in time and frequency domains was exploited. Under the hypothesis of Gaussianity of the observed data, we consider the zero-mean stationary bivariate stochastic process $S={[M F]}^{T}$, describing the dynamical activity of the beat-to-beat time series of MAP and MCBv. The interactions between the two individual processes $M$ and $F$ can be modelled by two auto- and cross-regressive (ARX) models whereby the present state of each process is regressed both on its own past and on the past of the other process, as follows [30]:

1 $$\left\{\begin{array}{l}M_n=\sum_{k=1}^p\;\alpha_{MM,k}M_{n-k}\;+\;\alpha_{MF,k}F_{n-k\;}+\;U_{\left.M\right|MF,n}\\M_n=\sum_{k=1}^p\;\alpha_{FM,k}M_{n-k}\;+\;\alpha_{FF,k}F_{n-k\;}+\;U_{\left.F\right|MF,n}\end{array}\right.$$

Equation 1a) is representative of the arm of the closed-loop system describing the coupled interactions between *M* and *F*, being $F$ the driver process and $M$ the target process. Conversely, Eq. (1b) is representative of the mechanism occurring along the opposite direction, i.e., the *pressure-to-flow* relationship, with ${M}$ the driver process and $F$ the target process.

In compact form, the vector AR model in Eq. (1) can be formulated as ${S}_{n}=\sum_{k=1}^{p}{A}_{k}{S}_{n-k}+ {U}_{n},$ where *p* is the model order, defining the maximum lag used to quantify interactions, $${A}_{k}=\left[\begin{array}{cc}{a}_{MM,k}& {a}_{MF,k}\\ {a}_{FM,k}& {a}_{FF,k}\end{array}\right]$$ is a 2 × 2 coefficient matrix quantifying the time-lagged interactions within and between the two processes at lag *k*, and $U_n= {[{U}_{M|MF,n}{ U}_{F|MF,n}]}^{T}$ is a vector of uncorrelated white noise processes with variances ${{\sigma }^{2}}_{M|MF}$ and ${{\sigma }^{2}}_{F|MF}$, respectively.

#### Time-domain and spectral measures of Granger Causality

Causality patterns were assessed through the well-known measure of Granger Causality (GC) [26], quantifying the improvement in predictability that the past states of a putative driver process bring to the present state of the target process above and beyond the predictability brought by the past states of the target itself [31]. Let us consider MCBv as the target process and MAP as the driver; the present state of the target $F$ is described first from the past of both *M* and *F* through the so-called *full* model defined in Eq. (1b), and then from the past of *F* only through the *restricted* AR model:

2 $${F}_{n}= \sum_{k=1}^{\infty }{b}_{FF,k}{F}_{n-k}+ {U}_{F|F,n},$$

where ${b}_{FF,k}$ is the model coefficient describing the interaction between ${F}_{n}$ and ${F}_{n-k}$ at lag *k*, and ${U}_{F|F,n}$ is a noise process with variance ${{\sigma }^{2}}_{F|F}$. Note that the order of the restricted AR model is theoretically infinite [32]; in practice, the model is implemented using *q* lags, with *q* sufficiently large. Assuming joint Gaussianity of the observed bivariate process $S,$ the predictability improvement yielded switching from the restricted to the full model can be quantified by the logarithmic measure of GC from *M* to *F* defined as [33]:

3 $${G}_{M\to F}=\mathrm{log}\frac{{{\sigma }^{2}}_{F|F}}{{{\sigma }^{2}}_{F|MF}}$$

In the case of joint Gaussian processes, the logarithmic GC measure is equivalent, up to a factor 2, to the information-theoretic measure of transfer entropy [27, 34]. The identification of the restricted model coefficients, ${b}_{FF,k}$, and the variance of the AR model residual, ${\sigma }_{F|F}^{2}$, is necessary to solve Eq. (3), involving a complex procedure starting from the computation of the covariance and the cross-covariance matrices between the present and the past variables of the two jointly Gaussian processes *M* and *F*, as detailed in [35]. To summarize, the procedure is based first on computing the autocovariance sequence of the bivariate process from its AR parameters via the well-known Yule-Walker equations, $\Gamma_k={\textstyle\sum_{l=1}^p}A_l\Gamma_{k-l}+\delta_{k0}\Sigma$ where $\Gamma_k$is the autocovariance matrix defined at lag *k* with $k=1,..,p-1$, ${A}_{l}$ is the AR coefficient matrix with $l=1,\dots ,p$, ${\delta }_{k0}$ is the Kronecker product and $\sum$ is the covariance matrix of the bivariate process [35]. The elements of $\Gamma_k$ are then rearranged for building the $q$ x $q$ covariance matrix of ${F}_{n}^{q}$, ${\sum }_{{F}_{n}^{q}}$, with ${F}_{n}^{q}=\left[{F}_{n-1}, \dots , {F}_{n-q}\right],$ and the 1 × *q* cross-covariance matrix of ${F}_{n}$ and ${F}_{n}^{q}$, ${\sum }_{{F}_{n}{F}_{n}^{q}}$. Specifically, these matrices are used to compute the restricted model coefficients vector ${B}_{FF}=[{b}_{FF,1}\dots {b}_{FF,q}]$ and the variance of the AR residuals ${\sigma }_{F|F}^{2}$ as (i) ${B}_{FF}= {\sum }_{{F}_{n},{F}_{n}^{q}}\cdot {\sum }_{{F}_{n}^{q}}^{-1}$ and (ii) ${\sigma }_{F|F}^{2}= {\sigma }_{F}^{2}- {\sum }_{{F}_{n},{F}_{n}^{q}}\cdot {\sum }_{{F}_{n}^{q}}^{-T}\cdot {\sum }_{{F}_{n},{F}_{n}^{q}}^{T}$, where ${\sigma }_{F}^{2}$ is the variance of *F*. Following the same rationale, the GC along the opposite arm, i.e., ${G}_{F\to M}$, can be computed to quantify the causal interaction from *F* to *M*.

To analyze causal interactions in the frequency domain, the model coefficients can be first represented through the Z- transform of (1), yielding $S\left(z\right)=H\left(z\right)U(z)$, where $H\left(z\right)={[I-\sum_{k=1}^{p}{A}_{k}{z}^{-k}]}^{-1}$ is the 2 × 2 transfer matrix, being *I* the 2 × 2 identity matrix. Computing $H(z)$ on the unit circle in the complex plane, the 2 × 2 power spectral density (PSD) matrix of the bivariate process is $P\left(\omega \right)=H(\omega )\sum {H}^{*}(\omega )$, where $\sum$ is the covariance matrix of $U$ and _*_ stands for Hermitian transpose [30]. This matrix contains the PSDs of *M* and *F* and the cross-PSDs between *M* and *F* as diagonal and off-diagonal elements, respectively. Under the hypothesis of strict causality leading to diagonality of $\sum$ [30, 36], the PSD of *F* can be factorized as:

4 $${P}_{F}\left(\omega \right)={\sigma }_{M|MF}^{2}{|{H}_{FM}(\omega )|}^{2}+{\sigma }_{F|MF}^{2}{|{H}_{FF}(\omega )|}^{2}$$

where ${\sigma }_{M|MF}^{2}{|{H}_{FM}\left(\omega \right)|}^{2}$ is the causal spectrum and ${\sigma }_{F|MF}^{2}{|{H}_{FF}\left(\omega \right)|}^{2}$ the non-causal spectrum of ${P}_{F}(\omega )$ [37]. Starting from the above factorization, the spectral GC can be defined as:

5 $${g}_{M\to F}\left(\omega \right)=\mathrm{log}\frac{{P}_{F}(\omega )}{{\sigma }_{F|MF}^{2}{|{H}_{FF}(\omega )|}^{2}}$$

quantifying at each frequency the portion of the target spectrum due only to the causal effects of the driver process [21, 37]. Remarkably, the spectral GC is linked to the time-domain GC defined in Eq. (3) by the spectral integration property:

6 $${G}_{M\to F}=\frac{1}{2\pi }{\int }_{-\pi }^{+\pi }{g}_{M\to F}(\omega )d\omega$$

Following the same rationale, the spectral GC along the opposite arm, i.e., ${g}_{F\to M}$, can be computed to quantify the contribution at each frequency of the causal interaction from *F* to *M* in the target spectrum.

#### Time-domain and spectral measures of Granger Autonomy

Patterns of self-dependencies were assessed through the measure of Granger Autonomy (GA) [26, 38], quantifying the predictability improvement brought to the present state of the target *F* by its own past states above and beyond the predictability brought by the past states of the driver *M* [39]. Operationally, GA is quantified comparing the *full* model defined in Eq. (1b) with a *restricted* cross-regressive (X) model, whereby $F$ is described only from the past of *M*:

7 $${F}_{n}=\sum_{k=1}^{\infty }{b}_{FM,k}{M}_{n-k}+{U}_{F|M,n}$$

where ${b}_{FM,k}$ is the model coefficient describing the interaction between ${F}_{n}$ and ${M}_{n-k}$ at lag *k*, and ${U}_{F|M,n}$ is a white noise process with variance ${{\sigma }^{2}}_{F|M}.$ Note that the order of the restricted AR model is theoretically infinite [32]; however, in practice the model is implemented using *q* lags, with *q* sufficiently large.

In analogy to Eq. (3), the predictability improvement is quantified by the logarithmic measure of GA given by:

8 $${G}_{F|M}=\mathrm{log}\frac{{\sigma }_{F|M}^{2}}{{\sigma }_{F|MF}^{2}}$$

which quantifies the strength of the autonomous dynamics of *F* comparing the error variances of the models in Eqs. (1b) and (7). For joint Gaussian processes, the concept of GA is equivalent, up to a factor 2, to the information-theoretic measure of conditional self-entropy, as described in [27]. The identification of the restricted X model parameters follows the same procedure as described in Sect. 2.2.1, with the difference that the model coefficients ${b}_{FM,k}$ in Eq. (7) are computed as ${B}_{FM}={\sum }_{{F}_{n},{M}_{n}^{q}}\cdot {\sum }_{{M}_{n}^{q}}^{-1}$, where ${B}_{FM}=[{b}_{FM,1}\dots {b}_{FM,p}]$, ${\sum }_{{M}_{n}^{q}}$ is the *q* x *q* autocovariance matrix of ${M}_{n}^{q}$ defined as ${\sum }_{{M}_{n}^{q}}=E[{M}_{n}^{q}{{M}_{n}^{q}}^{T}]$, ${\sum }_{{F}_{n,}{M}_{n}^{q}}$ is the cross-covariance matrix of ${F}_{n}$ and ${M}_{n}^{q}$ defined as ${\sum }_{{{F}_{n}M}_{n}^{q}}=E[{F}_{n}^{q}{{M}_{n}^{q}}^{T}]$. The variance of the residuals ${\sigma }_{F|M}^{2}$ in Eq. (7) is computed as ${\sigma }_{F|M}^{2}= {\sigma }_{F}^{2}- {\sum }_{{F}_{n},{M}_{n}^{q}}\cdot {\sum }_{{M}_{n}^{q}}^{-T}\cdot {\sum }_{{F}_{n},{M}_{n}^{q}}^{T}$. Following the same rationale, the self-dependencies of *M* can be assessed using the ${G}_{M|F}$ measure.

Similar to the description of causal interactions in Sect. 2.2.1, self-dependencies in the frequency domain are first obtained by describing the transfer function of the bivariate AR model formed by Eqs. (1a) and (7) in the Z domain as $S\left(z\right)=R\left(z\right)W(z)$, where W(z) is the Z-transform of the noise vector ${W}_{n}={[{U}_{M|MF,n}{U}_{F|M,n}]}^{T}$ and R(z) is the 2 × 2 transfer matrix computed as follows [27]:

9 $$R\left(z\right)=\left[\begin{array}{cc}{R}_{MM}(z)& {R}_{MF}(z)\\ {R}_{FM}(z)& {R}_{FF}(z)\end{array}\right]={\left[\begin{array}{cc}1-{A}_{MM}(z)& -{A}_{MF}(z)\\ -{B}_{FM}(z)& 1\end{array}\right]}^{-1}$$

with $${A}_{MM}\left(z\right)=\sum_{k=1}^{p}{a}_{MM,k}{z}^{-k}$$, $${A}_{MF}\left(z\right)$$$$=\sum_{k=1}^{p}{a}_{MF,k}{z}^{-k}$$, $${B}_{FM}\left(z\right)$$$$=\sum_{k=1}^{p}{b}_{FM,k}{z}^{-k}$$. $$R\left(z\right)$$ is then computed on the unit circle of the complex plane ($$z={e}^{j\omega }$$) to obtain the 2 × 2 complex transfer function in the frequency domain, $$R\left(\omega \right).$$ At this stage, we observe that in Eq. (7) the removal of the predictable self-dynamics of the target process increases the probability of being included in the residual $${U}_{F|M}$$, meaning that these are not modelled by the element $${R}_{FF(\omega )}$$ of the transfer function matrix $$R(\omega )$$. Accordingly, self-dependencies, modelled by $${H}_{FF}(\omega )$$, can be emphasized by comparing the transfer functions of the full and restricted model (i.e., $${H}_{FF}(\omega )$$ and $${R}_{FF}(\omega )$$, respectively) as follows:

10 $${g}_{F|M}\left(\omega \right)=\mathrm{log}\frac{{\sigma }_{F|M}^{2}{|{H}_{FF}(\omega )|}^{2}}{{\sigma }_{F|MF}^{2}{|{R}_{FF}(\omega )|}^{2}}$$

Remarkably, the spectral measure of self-dependencies defined in Eq. (10) is linked to the time-domain GA by the spectral integration property:

11 $${G}_{F|M}=\frac{1}{2\pi }{\int }_{-\pi }^{\pi }{g}_{F|M}\left(\omega \right)d\omega$$

Following the same rationale, the spectral GA along the opposite arm, i.e., ${g}_{M|F}$, can be computed to quantify the internal dependencies patterns of $F$.

### Data analysis

Standard time-domain statistical parameters such as the mean ($\mu$) and variance (${\sigma }^{2})$ were first computed on the *H*, *M* and *F* time series measured for each subject and experimental condition; the corresponding symbols and measurement units are ${\mu }_{H} [ms]$, ${\sigma }_{H}^{2}$ [${ms}^{2}]$, ${\mu }_{M} [mmHg]$, ${\sigma }_{M}^{2}$ [${mmHg}^{2}]$, ${\mu }_{F}$ [$cm\cdot {s}^{-1}$], ${\sigma }_{F }^{2}$[${cm}^{2}\cdot {s}^{-2}$].

Before computing the GC and GA measures, time series were pre-processed to remove the slow trends through an AR high-pass filter [40] (zero phase; cut-off frequency 0.0156 Hz) and the mean value.

GC and GA measures were then calculated on $M$ and $F$ time series for each subject in the two experimental conditions (*PRE*, *POST*), respectively regarded as realizations of the MAP and MCBv discrete-time processes. These processes were assumed as uniformly sampled with a sampling frequency equal to the inverse of the mean heart period (HP, i.e., ${f}_{s}=\frac{1}{HP})$.

**Fig. 1:**
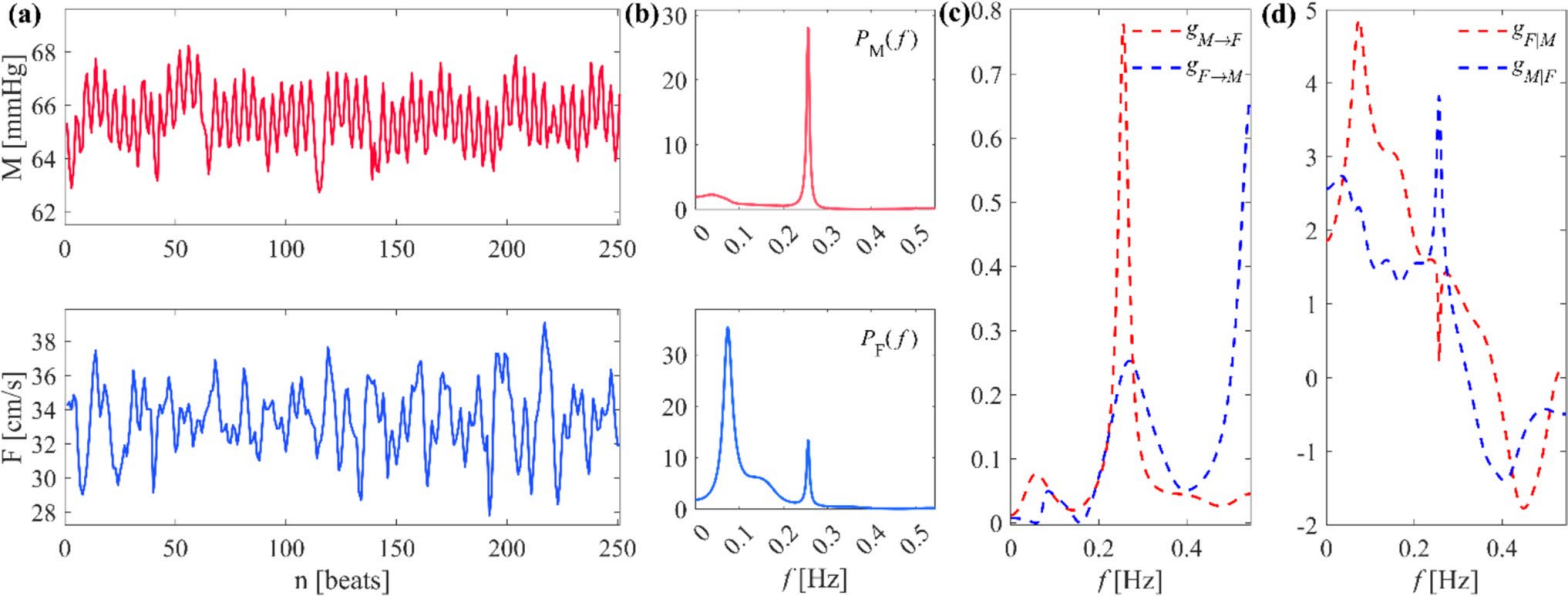
**(a)** Example of *M* (top), and *F* (bottom) time series for a representative subject in the *POST* condition, respectively representing MAP and MCBv; **(b)** power spectrum of *M*, ${P}_{M}(f)$ (top); power spectrum *F*, ${P}_{F}(f)$ (bottom); **(c)** measure of spectral Granger causality along the *pressure-to-flow* link, ${g}_{M\to F}$ (red dashed line), and along the *flow-to-pressure* link, ${g}_{F\to M}$ (blue dashed line); **(d)** measure of spectral Granger autonomy of the *F* process, ${g}_{F|M}$ (red dashed line), and of the *M* process, ${g}_{M|F}$ (blue dashed line)

A bivariate AR model in the form of Eq. (1) was fitted on each pair of pre-processed series using vector least squares identification and setting the model order *p* according to the multivariate version of the Akaike Information Criterion (AIC) [41] with maximum scanned model order equal to 14 [21]; the series and the PSD profiles were visually inspected and model orders were manually set when necessary, i.e., in case of too many (or too few) spectral peaks, according to physiological remarks and on the basis of previous studies [27]. After AR identification, the time-domain and spectral measures of GC and GA were computed and integrated within the three frequency bands of physiological interest for variability analysis, i.e., the very-low frequency (VLF, *f* ∈ [0.02 − 0.07] Hz), low frequency (LF, *f* ∈ [0.07 − 0.15] Hz) and high frequency (HF, *f* ∈ [0.15 − 0.4] Hz) bands of the spectrum [42]. The definition of frequency bands adopted in previous works [11, 43] was slightly varied according to the experimental context (i.e., mechanical ventilation during propofol general anaesthesia), centering the HF band on the mean ventilatory rate during anaesthesia to avoid respiratory peaks within LF band. An example of *M* and *F* time series, their power spectral densities and the spectral profiles of the GC and GA spectral measures for a representative subject is illustrated in Fig. 1.

### Surrogate and statistical data analysis

To test the statistical significance of the causality and autonomy measures (${g}_{M\to F}, {g}_{F\to M}$ and ${g}_{F|M}, {g}_{M|F} ,$ respectively) described in the previous Sects. 2.2.1 and 2.2.2, we used two different procedures for surrogate data generation according with previous works [27], specifically, the Iterative Amplitude Adjusted Fourier Transform (IAAFT) method for time and spectral measures of GC and the bootstrap method for time and spectral measures of GA. The IAAFT method, which represents an advancement over the Fourier transform (FT) algorithm [44], generates surrogate time series which preserve the individual linear correlation properties of two series but destroy any correlation between them [45]. On the other hand, the bootstrap method, applied for GA measures, uses explicit model equations extracted from the data to generate surrogates that satisfy the null hypothesis of absence of internal dynamics (H_0_) [46]; specifically, each original *M* series was fitted with the ARX model defined in Eq. (1a), while the corresponding *F* series was fitted with the X model defined in Eq. (7) to test H_0_. Finally, pairs of surrogate time series were generated feeding the models with noise realizations obtained randomly shuffling the samples of the estimated residuals, as described in [27].

Three-hundred pairs of surrogate time series were generated for each subject and condition by iterating these procedures, and the time-domain and spectral measures of GC and GA were computed at each iteration. The significance of the measures, computed either in the time domain or integrating the spectral functions over the VLF, LF or HF bands, was assessed comparing the values obtained on the original time series with the confidence limits of the surrogate distribution (with 5% significance). Specifically, the time and spectral measures of GC were deemed as statistically significant if their value was respectively above the 95*th* percentile of the GC surrogate distribution, while the time and spectral measures of GA, as they can take both positive and negative values, were deemed as statistically significant if their value was respectively above the 97.5*th* or below the 2.5*th* percentile of the GA surrogate distribution.

Moreover, the distributions of the time-domain markers and of GC and GA measures computed across subjects were tested for normality using the Anderson–Darling test [47]. Since the hypothesis of normality was rejected for most distributions, and given the small sample size, the paired Wilcoxon signed rank test was employed to assess the statistical significance of the differences of each index between conditions (*PRE* vs *POST*) [48] with a significance level of 5%.

## Results

Table 2 reports the time domain parameters computed on the *H*, *M* and *F* time series (mean and standard deviation labeled respectively as ${\mu }_{H}$,${\sigma }_{H}^{2}$,${\mu }_{M}$,${\sigma }_{M}^{2}$,${\mu }_{F}$, ${\sigma }_{F}^{2}$) during both *PRE* and *POST* experimental conditions. The values are reported as mean ± standard deviation (SD).

**Table 2:** Time domain markers (mean µ and variance ${\sigma }^{2}$) computed on H, M and F series (${\mu }_{H}$, ${\sigma }_{H}^{2}$; ${\mu }_{M}$, ${\sigma }_{M}^{2}$; ${\mu }_{F}$, ${\sigma }_{F}^{2}$, respectively) during the *PRE* and *POST* experimental conditions. The symbol * indicates p < 0.05 *POST* versus *PRE*, Wilcoxon test

Parameter	*PRE*	*POST*
${\mu }_{H}[ms]$	901.45 ± 140.55	1029.4 ± 128.81*
${\sigma }_{H}^{2}$[${ms}^{2}]$	883.67 ± 903.57	338.81 ± 512.15*
${\mu }_{M}[mmHg]$	100.70 ± 13.10	69.26 ± 7.62*
${\sigma }_{M}^{2}[{mmHg}^{2}]$	10.25 ± 4.03	3.36 ± 2.20*
${\mu }_{F}[cm \cdot {s}^{-1}]$	47.46 ± 18.46	35.99 ± 10.22
${\sigma }_{F}^{2}[{cm}^{2}\cdot {s}^{-2}]$	17.01 ± 13.87	4.71 ± 2.98*

Results indicate a statistically significant increase of ${\mu }_{H}$ during *POST,* while ${\sigma }_{H}^{2}$, ${\mu }_{M}$, ${\sigma }_{M}^{2}$, ${\sigma }_{F}^{2}$ decreased significantly. Only ${\mu }_{F}$ does not exhibit a statistically significant change.

**Fig. 2:**
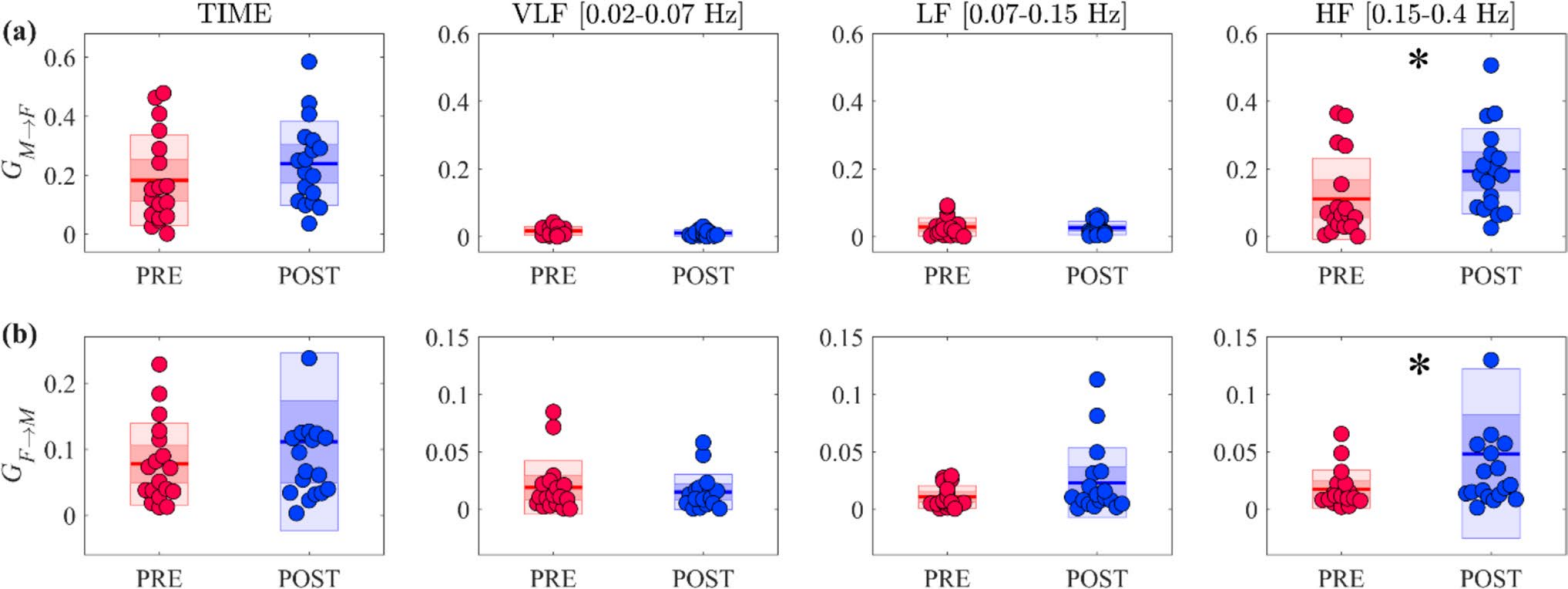
Time and frequency domain causal analysis of cerebrovascular time series. Plots depict the boxplot distributions and individual values of GC measures computed along **(a)** the *pressure-to-flow* link (${G}_{M\to F,}$ top row) and **(b)** the *flow-to-pressure* link (${G}_{F\to M},$ bottom row) in the time domain (first column of each subplot) and integrating the spectral functions within the VLF, LF and HF frequency bands (second, third and fourth columns of each subplot). Measures were evaluated in the *PRE* (red) and *POST* (blue) conditions. In all panels, horizontal lines represent mean values, while darker and lighter colour shades delimit one standard deviation and 95% confidence interval, respectively. Statistically significant differences (p < 0.05): *, *PRE* vs *POST*, Wilcoxon signed rank test for paired data

The results of time and spectral Granger causality measures are depicted in Fig. 2. In the time domain, no significant changes were detected across the two experimental conditions (Fig. 2 left plots). On the other hand, the evaluation of GC in bands of physiological interest (i.e., VLF, LF and HF) highlighted a statistically significant increase of ${G}_{M\to F}$ and of ${G}_{F\to M}$ in the HF band in *POST* condition if compared to *PRE*.

**Fig. 3:**
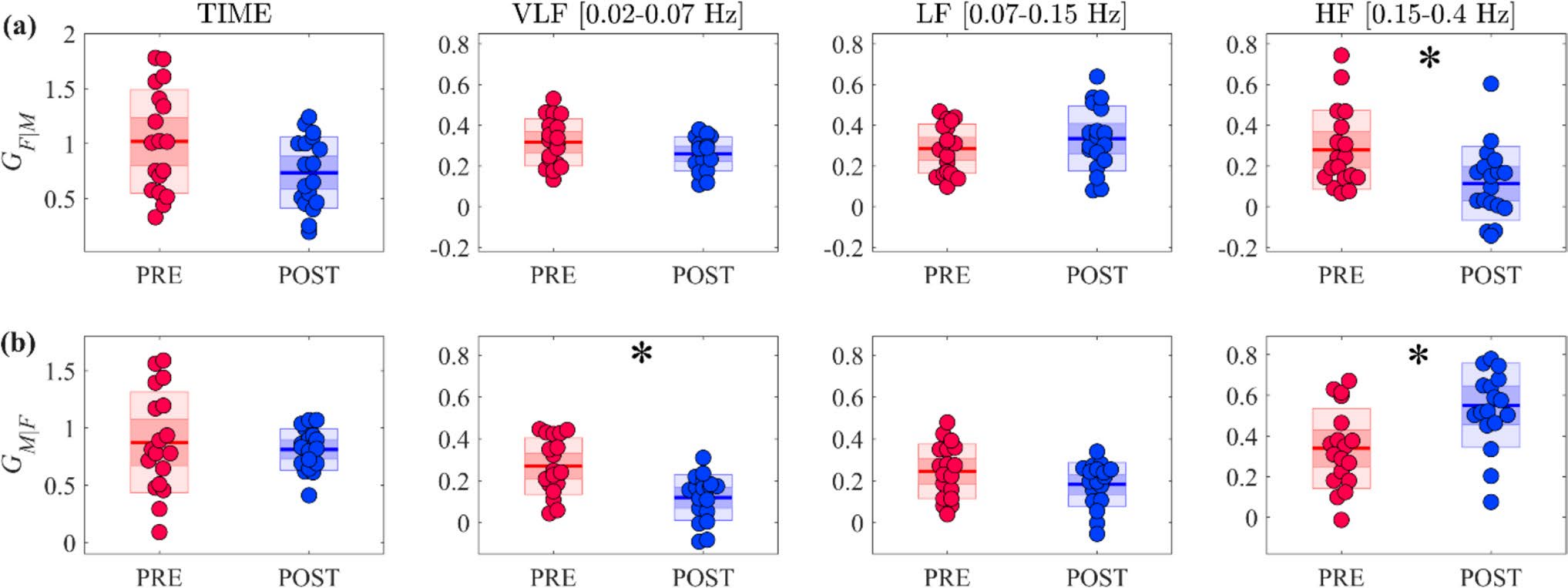
Time and frequency domain analysis of the self-dynamics of cerebrovascular time series. Plots depict the boxplot distributions and individual values of GA measures computed for **(a)** the *F* process (${G}_{F|M,}$ top row) and **(b)** the *M* process (${G}_{M|F},$ bottom row) in the time domain (first column of each subplot) and integrating the spectral functions within the VLF, LF and HF frequency bands (second, third and fourth columns of each subplot). Measures were computed in the *PRE* (red) and *POST* (blue) experimental conditions. In all panels, horizontal lines represent mean values, while darker and lighter colour shades delimit one standard deviation and 95% confidence interval, respectively. Statistically significant differences (p < 0.05): *, *PRE* vs *POST*, Wilcoxon signed rank test for paired data

The results of the time and spectral analysis of GA are depicted in Fig. 3. Neither of the time-domain measures show significant changes between the two experimental conditions (Fig. 3 left plots). On the other hand, the evaluation of spectral GA highlights in *POST* a decrease of ${G}_{F|M}$ in the HF band and of ${G}_{M|F}$ in the VLF band and an increase of ${G}_{M|F}$ in the HF band.

**Fig. 4:**
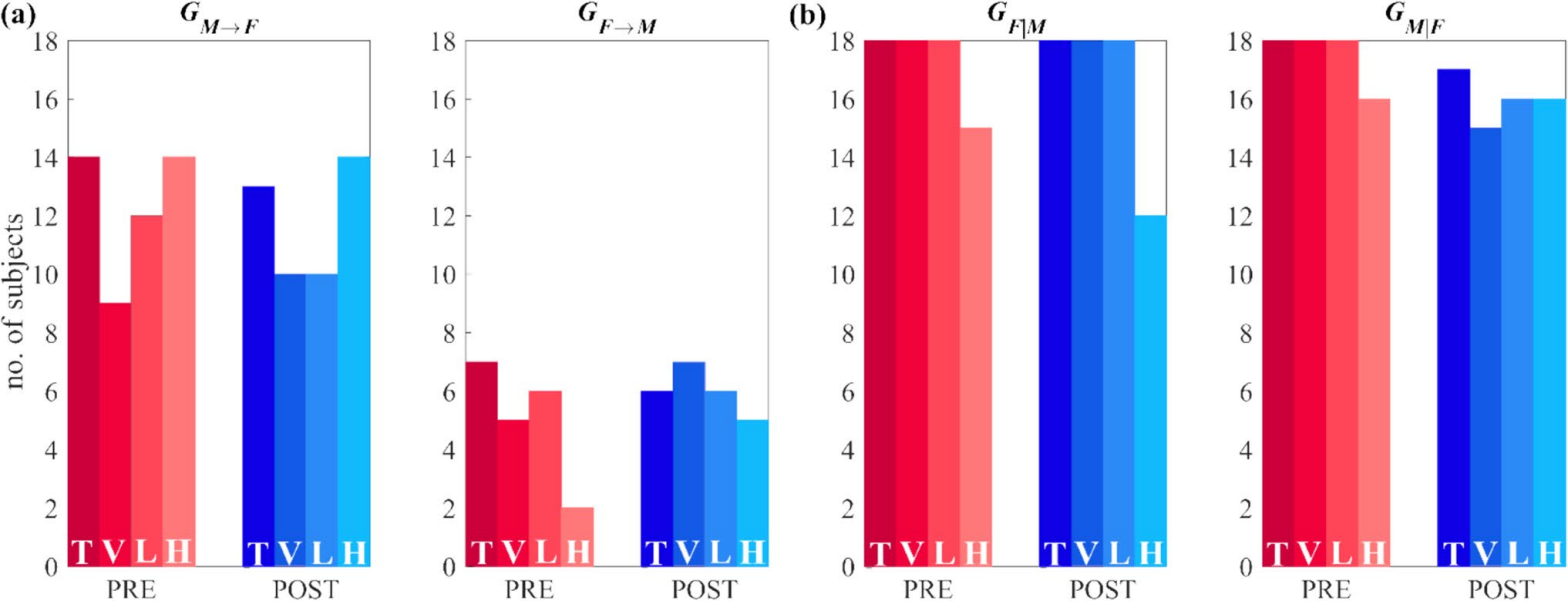
Surrogate data analysis for causal and autonomous dynamics measures. Barplots depict the number of subjects (out of 18) for whom the measures of **(a)** GC (${G}_{M\to F}$, ${G}_{F\to M})$ and **(b)** GA (${G}_{F|M}$, ${G}_{M|F}$) along the *pressure-to-flow* and the *flow-to-pressure* links respectively deemed as statistically significant according to surrogate data analysis. The letter within a bar indicates the specific measure (T = time domain; V = spectral measure in VLF band; L = spectral measure in LF band; H = spectral measure in HF band), while the colour represents the condition (*PRE*, red bars; *POST*, blue bars)

Figure 4 shows the results of surrogate data analysis on the causality and autonomy measures evaluated in *PRE* and *POST* conditions. As reported, the number of subjects exhibiting statistically significant values is higher for autonomy measures than for causality measures.

## Discussion

### Time domain markers

The trends of the time domain markers reported in Table 2 document the cerebrovascular response to general anaesthesia with propofol in patients undergoing CABG surgery. Specifically, the increase of ${\mu }_{H}$ [49, 50] is indicative of the sympathetic withdrawal after the application of standard premedication in the *PRE,POST* protocol [28], whereas the decrease of ${\sigma }_{H}$ is a consequence of the depression of autonomic function [51, 52]. Additionally, as widely documented in previous studies [17, 28, 29, 53], the significant reduction of ${\mu }_{M}$ reflects the effect of the vasodilation and the decrease of peripheral resistances, and can be also linked to the possible effect of positive pressure mechanical ventilation, which can affect venous return increasing intrathoracic pressure [54]. The decline of ${\sigma }_{M}^{2}$ can be primarily the consequence of sympathetic inhibition due to the impact of propofol on autonomic regulation [55]. During propofol general anaesthesia, autonomic depression is mainly the result of reduced sympathetic control leading to reduced ventricular contractility and reduction of peripheral vascular resistances [15]. However, the decrease of ${\sigma }_{M}^{2}$ could also be related to the possible effects of mechanical ventilation on blood pressure variability, with the change in thoracic pressure cyclically affecting blood pressure (mainly via venous return). In addition, the effect of variation in respiratory sinus arrhythmia could not be excluded, given that the lower respiratory rates when spontaneously breathing (in *PRE*) produce a higher RSA [56]. As known from previous studies [28], among anaesthetics agents propofol has the well-known property of preserving CA, maintaining the cerebral perfusion stable despite changes in systemic blood pressure. In this regard, the not statistically significant decrease of ${\mu }_{F}$ suggests that cerebrovascular regulation is compensating to some extent the spontaneous drops in MAP, confirming previous studies indicating that propofol preserves dynamic CA [29, 57, 58]. This finding is particularly important because it corroborates the ability of propofol to support cerebrovascular stability during anaesthesia, thereby reducing the risk of adverse neurological outcomes after CABG surgery. Conversely, previous works with the same protocol reported a significant decrease in ${\mu }_{F}$ following the induction of propofol general anaesthesia as a consequence of a reduced metabolic oxygen rate [17, 29]. In our work, the effect of oxygen rate consumption could not be ruled out, since the *p* value of the statistical comparison between *PRE* and *POST* with regard to ${\mu }_{F}$ (*p* = 0.0582) is close to the significance threshold and given also the statistically significant decrease of ${\sigma }_{F}^{2}$ in *POST*. However, the variability of the result may be influenced by the operator’s experience and skill in using TCD to detect CBv from the middle cerebral artery, as TCD is inherently operator-dependent [59–61].

### Causality and autonomy measures

The analysis of GC and GA measures in time and spectral domains (Fig. 2 and Fig. 3) revealed a predominance of autonomous dynamics mechanisms compared to causal interactions in cerebrovascular system, demonstrated by the higher values of GA compared to GC measures along the *pressure-to-flow* and *flow-to-pressure* links, also corroborated by the very high number of subjects for whom the GA measures were deemed as statistically significant (Fig. 4b). Moreover, with regard to causal interactions (Fig. 2), our results evidenced both in *PRE* and *POST* conditions a preponderance of GC measures along the *pressure-to-flow* link, especially in HF band. This is once again corroborated by the higher number of subjects for whom the GC measures along the *pressure-to-flow* link were deemed as statistically significant than on the *flow-to-pressure* link (Fig. 4a).

The invariance of time domain indices of GC and GA across the analysed experimental conditions and in both investigated relations, i.e., *pressure-to-flow* and *flow-to-pressure* links (Fig. 2 and Fig. 3, left plots), suggests the overall high regulatory capacity of the cerebrovascular system in response to internal or external stimuli in patients undergoing CABG surgery. On the other hand, the evaluation of the same measures within the frequency bands of physiological interest for this application highlights some evident variations between the two experimental conditions. These different behaviours of time-domain and spectral measures confirm the importance of assessing causal and autonomy patterns within specific bands in the frequency domain to capture mechanisms that could remain hidden if only a whole-band time-domain analysis is performed.

Specifically, while the spectral GC in the VLF and LF bands was not modulated significantly by the induction of anaesthesia, moving from *PRE* to *POST* a statistically significant increase of spectral GC was documented along the *flow-to-pressure* and *pressure-to-flow* links in HF band. This increase, which indicates higher coupling between the HF oscillations of MAP and MCBv, is likely related to the simultaneous effect induced by mechanical breathing on both variability series during general anaesthesia. Indeed, respiration is known to be a confounder or suppressor of the closed-loop relationship responsible for cerebrovascular dynamic interactions, as assessed by the spontaneous variability of MAP and MCBv [18]. Its effect could generate regular oscillations in both time series at the rate of mechanical ventilation, leading to an increase in the predictability of *M* from *F* and of *F* from *M* [62–64]. More specifically, the ability of MCBv in predicting MAP might be related to the depression of the sympathetic control induced by propofol, that might affect the efficiency of the *flow-to-pressure* link. These findings may suggest that respiration could be a confounder for the Cushing’s reflex as documented in other studies that condition on breathing [18]. Likewise, the increase of spectral GC along the *pressure-to-flow* link in the HF band reflects the same effect of the mechanical respiratory rate on which the HF band is approximately centred, inducing a stronger dependence of *F* dynamics on *M* as a result of the causal information transferred by the past of the driver *M* under the alleged effect of positive pressure ventilation in *POST* [65, 66]. Therefore, we hypothesise that the disturbing action of mechanical breathing may be sufficiently powerful on our pathological population in altering the relationship between blood pressure and cerebral blood flow [29].

As regards autonomy measures (Fig. 3), they were strong and statistically significant in the majority of subjects in both experimental conditions and in all frequency bands, documenting the existence of relevant internal dynamics in the regulation of MAP and MCBv. Nevertheless, the spectral analysis evidences a progressive reduction of ${G}_{F|M}$ computed in the HF band, also mirrored by a decrease in the number of subjects showing significant GA. Methodologically, the decrease of spectral GA indicates a reduced impact of self-regulatory mechanisms that operate regardless of the MCBv-MAP interactions during *POST*. More specifically, weak internal dynamics reflect a decrease in the ratio of the two transfer functions of the full and restricted models, meaning lower values of ${|H}_{FF}\left(\omega \right)|$ ^2^ compared to ${|R}_{FF}\left(\omega \right)|$ ^2^. The decrease in the amplitude of ${|H}_{FF}\left(\omega \right)|$ ^2^ may indicate that the internal flow dynamics in HF lose significance, indicating that the oscillation either moves to other neighbouring bands or that its power decreases [21]. On the contrary, the significant increase of ${G}_{M|F}$ in the HF band suggests that the internal regulatory mechanisms of MAP gradually become stronger in *POST*, inducing high values in the amplitude of the transfer function of the full model, ${|{H}_{MM}(\omega )|}^{2}$, indicative of an increase in the internal MAP dynamics in HF. From a physiological point of view, ${|H}_{FF}\left(\omega \right)|$ and ${|H}_{MM}\left(\omega \right)|$ measure the intrinsic ability of MAP and MCBv to self-regulate themselves regardless of their interactions. ${|H}_{MM}\left(\omega \right)|$ might be inflated by modifications of vascular properties of the arterial tree, for example departures from a purely resistance behaviour to a more capacitive one, and ${|H}_{FF}\left(\omega \right)|$ by longer memory effects in MCBv regulation. However, even factors unaccounted for by the adopted model of the interactions between MAP and MCBv might play a role because any influences that are not explicitly described by the model might modify GA. As a consequence, being respiration an exogenous factor that drives variability of MAP and MCBv directly, regardless the interactions between them, it is not surprising to find out that, in presence of a stronger mechanical drive imposed by mechanical ventilation, GA indexes could be modified. These results reveal a complementary behaviour of GA in the HF band, with an increased contribution of autonomous dynamics when *M* is considered as the target process, while a decreased one when *F* is the target process. A possible physiological explanation of this different behaviour lies in the effects of mechanical ventilation, in addition to the action of propofol-based general anaesthesia on respiration: it is likely that the mechanical respiratory drive on MAP becomes stronger during anaesthesia, thus inducing a more evident oscillation in MAP that is not explained by MCBv, thus increasing the GA of MAP given MCBv; at the same time, the respiration-related oscillations of MCBv are more explained by MAP during anaesthesia and thus become less self-predictable, resulting in a decrease of the GA index. Among the factors that might alter GA markers there is the partial pressure of carbon dioxide (PaCO_2_) that is a well-known confounding factor for MCBv regulation given that an increase of PaCO_2_ above the physiological value produces cerebral vasodilation, while a decrease induces vasoconstriction. Since PaCO_2_ punctual measures were available only in *POST* [29], PaCO_2_ dynamics cannot be included in the model of the MCBv-MAP interactions [21] and, thus the possible influences of PaCO_2_ dynamics cannot be conditioned out in the *PRE*-*POST* comparison. Therefore, it cannot be excluded that the observed modifications of the GA indexes could be due modifications of PaCO_2_. However, since the modifications of GA indexes occur in the HF band it is extremely unlikely the slow dynamical variations of PaCO_2_ could play a role. Conversely, PaCO_2_ might be responsible for biasing ${G}_{M|F}$ in the VLF band and could explain the observed variations of ${G}_{M|F}$.

## Conclusion

This work assessed causal and autonomy patterns of the closed-loop cerebrovascular interactions in patients scheduled for CABG surgery, evaluated before and after administration of propofol general anaesthesia and mechanical ventilation, using time and spectral formulation of GC and GA measures. Our results evidenced that time-domain indices remain consistent across experimental conditions in both the *pressure-to-flow* and *flow-to-pressure* links, indicating strong cerebrovascular regulatory capacity in patients undergoing surgery. Further insights were revealed by the frequency analysis, since spectral GC allowed to evidence in post-anaesthesia a greater cerebral blood flow dependence on arterial pressure and vice versa, as well as the effect of the mechanical ventilation in HF. Spectral GA allowed to infer a weakened internal regulation of MCBv in the HF band and of MAP in the VLF band, possibly related to modifications of PaCO_2_, and an enhanced autonomous regulation of MAP in HF. Overall, our findings highlighted the benefit of the combined use of GC and GA measures in efficiently characterizing the causality and self-dependencies patterns of the closed-loop system and the need for spectral analysis to capture subtle cerebrovascular dynamics, as time-domain measures alone may overlook some mechanisms affecting cerebral perfusion and autoregulation.

Future works should confirm the obtained findings on a larger and gender-balanced dataset, in order to take into account also possible gender differences in cerebrovascular regulation. Further studies should be also designed to extend to the multivariate case the measures of GC and GA in both time and frequency domains, i.e., analysing higher order interactions involving three or more processes according to Network Physiology approach [67, 68]. This could be useful to overcome current limitations of our measures taking also into account the effects of unobserved confounding factors (e.g., respiration and/or PaCO_2_) in the assessment of the dynamics of interactions between the cerebral blood flow and the arterial blood pressure, providing new insights into the regulatory mechanisms of cerebrovascular networks.

## Declarations

### Data availability

Data used in the present study are available from the authors on reasonable request.
